# The calcar screw in angular stable plate fixation of proximal humeral fractures - a case study

**DOI:** 10.1186/1749-799X-6-50

**Published:** 2011-09-24

**Authors:** Georg Osterhoff, Christian Ossendorf, Guido A Wanner, Hans-Peter Simmen, Clément M Werner

**Affiliations:** 1 Division of Trauma Surgery, University Hospital Zurich, Rämistrasse 100, 8091 Zurich, Switzerland

**Keywords:** Proximal humerus, fracture, locked screw, locking plate

## Abstract

**Background:**

With new minimally-invasive approaches for angular stable plate fixation of proximal humeral fractures, the need for the placement of oblique inferomedial screws ('calcar screw') has increasingly been discussed. The purpose of this study was to investigate the influence of calcar screws on secondary loss of reduction and on the occurrence of complications.

**Methods:**

Patients with a proximal humeral fracture who underwent angular stable plate fixation between 01/2007 and 07/2009 were included. On AP views of the shoulder, the difference in height between humeral head and the proximal end of the plate were determined postoperatively and at follow-up. Additionally, the occurrence of complications was documented. Patients with calcar screws were assigned to group C+, patients without to group C-.

**Results:**

Follow-up was possible in 60 patients (C+ 6.7 ± 5.6 M/C- 5.0 ± 2.8 M). Humeral head necrosis occurred in 6 (C+, 15.4%) and 3 (C-, 14.3%) cases. Cut-out of the proximal screws was observed in 3 (C+, 7.7%) and 1 (C-, 4.8%) cases. In each group, 1 patient showed delayed union. Implant failure or lesions of the axillary nerve were not observed. In 44 patients, true AP and Neer views were available to measure the head-plate distance. There was a significant loss of reduction in group C- (2.56 ± 2.65 mm) compared to C+ (0.77 ± 1.44 mm; p = 0.01).

**Conclusions:**

The placement of calcar screws in the angular stable plate fixation of proximal humeral fractures is associated with less secondary loss of reduction by providing inferomedial support. An increased risk for complications could not be shown.

## Background

Patients with minimally displaced or stable fractures of the proximal humerus are treated conservatively in the majority of cases [1]. In contrast, patients with fractures fulfilling the criteria of instability, referred to as displaced or unstable fractures, benefit from surgical intervention which mostly renders reliable results, both, clinically and radiographically [2,3]. However, surgery of displaced proximal humeral fractures is technically demanding. A wide array of surgical options has been described and controversially discussed [4-10].

The introduction of locking plate systems represents a milestone in fracture treatment with the advantage of improved osseous anchorage and higher resistance to failure by combining axial and angular stability [11,12]. These plates are suitable for pathologic and osteoporotic fractures. Additionally, locking plates do not depend on friction or compression between plate and bone to stabilize the fracture and therefore do not compromise periosteal blood supply [13,14].

**Figure 1:**
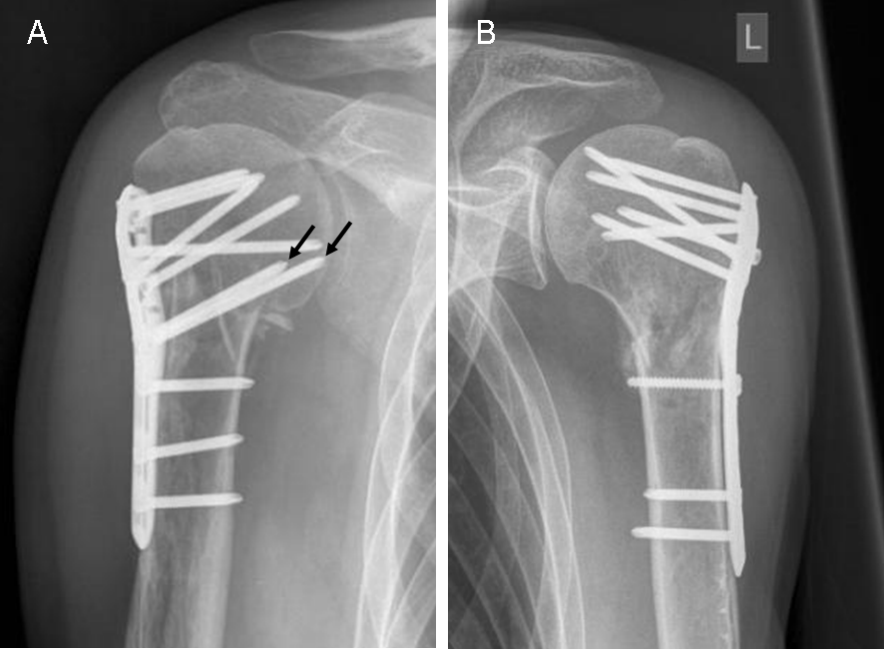
**Angular stable plate fixation with (A) and without (B) calcar screws**. Arrows pointing at calcar screws.

In proximal humeral fractures, the particular proximity of tendinous and neurovascular structures of the joint and the characteristic bone strength distribution of the humeral head [15] require a fixation system with predetermined screw settings. The Philos plate system (Synthes, Oberdorf, Switzerland) was developed to meet these requirements by using a tridimensionally-fashioned locking system for the proximal screws. However, several studies with short- to mid-term experiences after Philos plate fixation suggest that-in spite of good overall clinical results-the implant's stiffness might lead to a higher rate of screw cut-out or cut-through [16-27]. A lack of medial support was suggested to be one possible reason [28]. In addition, the presence or absence of medial support was described as a significant predictor of loss of fracture reduction [29]. One simple way of gaining medial support is the insertion of one or two screws running tangentially to the medial curvature of the humeral surgical neck (calcar screws, Figure 1). Yet, with new minimally-invasive approaches for the angular stable plate osteosynthesis the need for these calcar screws has increasingly been discussed. It has been suggested that the proximity to the anterior [30] and posterior [31] circumflex arteries might compromise perfusion of the humeral head and by this lead to delayed-union or non-union or to osteonecrosis. As they are supposed to additionally stiffen the osteosynthetic construct, calcar screws may also increase the risk of cut-out [32].

Therefore, orthopaedic surgeons cannot be sure if they either increase the risks of complications or potentially miss a better long term fracture reduction implicating a better treatment outcome.

Thus, the purpose of this study was to investigate if the presence of calcar screws can reduce secondary loss of reduction and if it has influence on the occurrence of possible complications-especially cut-out and axillary nerve lesions.

## Methods

All patients with a proximal humeral fracture who underwent angular stable plate fixation (PHILOS, Synthes, Oberdorf, Switzerland) in our hospital between 01/2007 and 07/2009 were included in the present study. All data was collected according to the terms of reference specified by the local ethics committee.

Criteria for exclusion were: age younger than 18 years, previous ipsilateral fractures of the humerus and bony metastases. The indication for surgery was set when posttraumatic radiographs showed evidence of displacement of > 1 cm or an angulation > 45° according to Neer's criteria for displaced fractures [33]. Fracture morphology was classified in two-, three- and four-part fractures on posttraumatic true AP and Neer radiographs. Surgery was performed either via a deltopectoral approach or in minimally-invasive technique via short delta-split approach combined with skin incisions for the distal screws, depending on the surgeons' choice.

All patients underwent a standardized postoperative treatment schedule characterized by early passive motion under physiotherapeutic surveillance.

**Figure 2:**
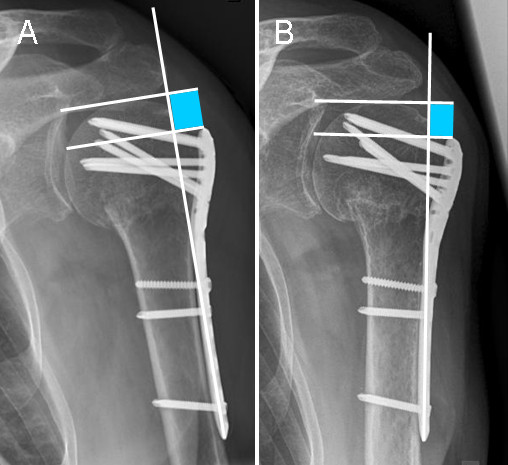
**Method of measuring the distance between humeral head and the proximal end of the plate (A) postoperatively and (B) after follow-up (as previously described by Gardner et al**.)

Differences in height between humeral head and the proximal end of the plate were determined on true AP radiographs of the shoulder, postoperatively and at follow-up, as described previously [29] (Figure 2). The distance between two lines orthogonal to the plate axis was measured, one line running through the proximal end of the plate and one through the tip of the humeral head. All measurements were performed by the first author using a digital caliper tool of the standard viewer software of our institution (Agfa Study Viewer 5.0.1, Agfa HealthCare, Mortsel, Belgium). An average value of 3 measurements of each radiographic distance was computed. A decrease was interpreted as a loss of reduction. Subsequently, the presence of screws running tangentially to the medial curvature of the humeral surgical neck (calcar screws) was determined (Figure 1).

Patients with one or two calcar screws were assigned to group C+, patients without a calcar screw to group C-. The surgical reports of all patients were checked for the approach that was used. Complications were evaluated based on follow-up radiographs (AP and Neer) and a retrospective chart review of the patients' medical records. The incidence of humeral head necrosis, delayed union, implant failure or neurological deficits was documented. Cut-out was defined as penetration of the proximal screws (humeral head screws) into the joint cavity in the absence of humeral head necrosis. Humeral head necrosis was determined by a collapse of the humeral head with an unrounding of the articular surface.

### Statistical Analysis

Statistical analysis of nominal data was done using 2-sided Fisher's Exact Tests, and metric data was processed using the Mann-Whitney Test with SPSS for windows 17.0 (SPSS, Chicago, Illinois, USA). Differences were considered significant for values of *p *< 0.05. A post-hoc power analysis for comparing loss of reduction was calculated using PS Power and Sample Size Calculations 3.0 (alpha error: 0.05) [34].

## Results

**Figure 3:**
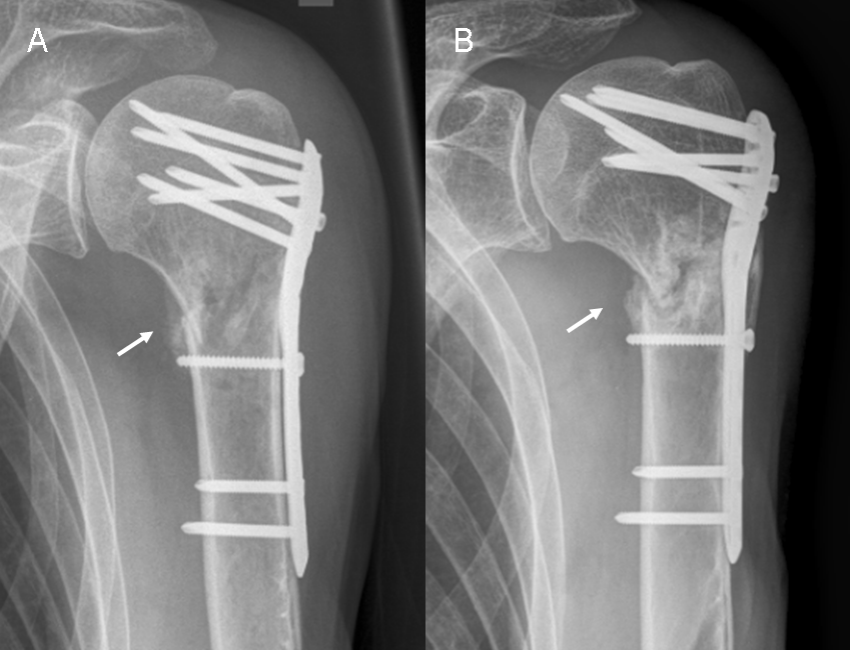
**Example of a failed plate fixation without calcar screws at 6 weeks (A) and 9 months (B) after surgery**. Notice non union at the medial cortex (white arrow).

**Figure 4:**
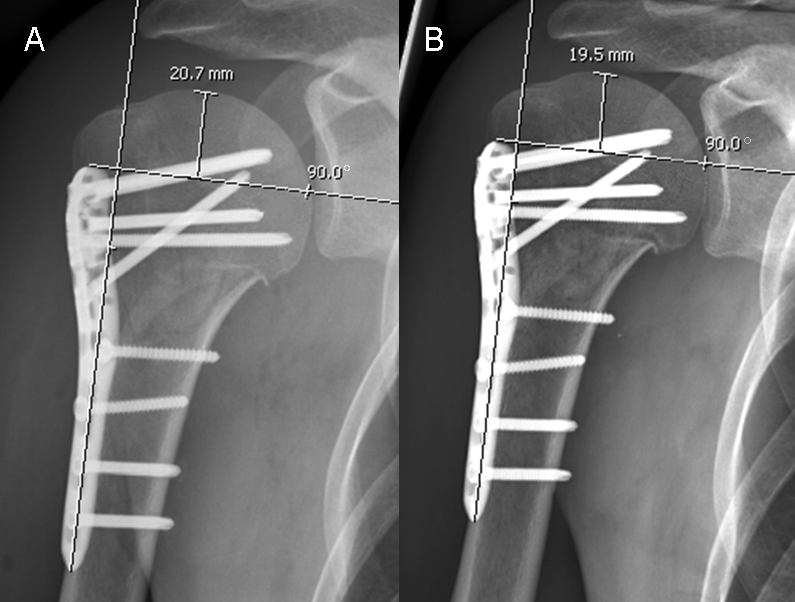
**Example of a patient in group C- (no calcar screw) with a loss of reduction of 1.2 mm when comparing postoperative (A) and follow-up radiographs at 3 months (B)**.

A total of 68 patients with proximal humeral fracture underwent angular stable plate fixation within the observation period. One patient died shortly after surgery because of non-related diagnoses. Six patients were lost to follow-up as they did not appear at their outpatient-clinic appointments for unknown reasons. One patient (group C+) presented with an early wound infect which made it necessary to remove the plate just 13 days postoperatively. Thus, follow-up was possible only in 60 patients (mean age: 57.9 ± 17.5 years). Twenty-one patients formed group C- (mean age 54 ± 20). Thirty nine patients formed the Group C+ (mean age 60 ± 16). A short delta-split (minimally-invasive) approach was used in twelve patients (57.1%) of group C- but in only one patient (2.6%) of group C+. Mean follow-up was 6.1 ± 4.8 months (range C+ 6.7 ± 5.6 months/C- 5.0 ± 2.8 months). Out of these, humeral head necrosis occurred in 6 (15.4%) cases in patients with calcar screws and 3 (14.3%) without calcar screws (p = 1). It could be noticed that fracture morphology differed between both groups and group C+ included considerably more complex fractures (Table 1). Head necrosis, in fact, was seen only in three- or four-part fractures. Cut-out of the proximal screws (Figure 3) was observed in 3 (C+, 7.7%) and in 1 (C-, 4.8%) cases (p = 1). In each group one patient showed delayed fracture union (p = 1). Implant failure or loosening of the screw heads in the plate was not observed. Revision surgery due to the complications named above was required in 6 (C+, 15.4%) and 4 (C-, 19.0%) patients (Table 2). No neurological deficits were observed in group C-, while in group C+ one patient had persistent dysaesthesia in his palm, most likely because of intraoperative stretch of the brachial plexus. Another patient in group C+ complained about paresthesia in all fingers of the operated arm although an electroneuromyography revealed no traceable nerval lesion and his underlying schizophrenic disease might have influenced the patient's perception. There was no clinical indication of a lesion to the axillary nerve in any of the 60 patients (Table 3). The measurement of the head-plate distance was only possible in 44 patients (C-: n = 16, C+: n = 28) due to incorrect projection of the radiographs in 16 patients. Measurements of head-plate distance (Figure 4) yielded a significant loss of reduction in group C- (2.56 ± 2.65 mm) compared to C+ (0.77 ± 1.44 mm; p = 0.01). Post-hoc analysis revealed a power of 0.97 for measurements of a loss of reduction (n = 44).

**Table 1:** Fracture morphology

	2 part	3 part	4 part	Total
**Calcar +**				
**N**	7	14	18	39
**%**	17.9	35.9	46.2	100

**Calcar -** (n = 21)				
**N**	8	8	5	21
**%**	38.1	38.1	23.8	100

**Total** (n = 60)				
**N**	15	22	23	60
**%**	25.0	36.7	38.3	100

**Table 2:** List of patients that required revision surgery

Patient		1^st ^- 2^nd^	Group	Approach	Complication	Intervention
**SO**,	**35 y**	8 w	C-	delt.-pect.	head necrosis	implant removal
**CG**,	**57 y**	12 w	C-	mipo	head necrosis	screw replacement
**CP**,	**77 y**	36 w	C-	mipo	head necrosis	implant removal
**BB**,	**81 y**	8 w	C-	mipo	l o r w/cut-out	screw replacement

**NU**,	**49 y**	51 w	C+	delt.-pect.	head necrosis	arthroplasty
**AH**,	**73 y**	20 w	C+	delt.-pect.	l o r w/cut-out	implant removal
**WL**,	**70 y**	16 w	C+	delt.-pect.	head necrosis	arthroplasty
**ED**,	**58 y**	8 w	C+	delt.-pect.	l o r w/cut-out	screw replacement
**WB**,	**52 y**	7 w	C+	delt.-pect.	head necrosis	head resection
**JJ**,	**68 y**	16 w	C+	delt.-pect.	l o r w/cut-out	screw replacement

**1^st ^- 2^nd^**: time between fracture fixation and secondary intervention. **y**: years. **w**: weeks.

**delt.-pect**.: deltopectoral approach. **mipo**: minimally-invasive short delta split approach.

**l o r w/cut-out**: loss of reduction with cut-out of the proximal screws.

**Table 3:** Complications and Reoperations due to complications

	Head necrosis	Delayed union	Cut-Out/-Through	Neurological deficits	Second surgery
**Calcar +** (n = 39)					
**N**	6	1	3	2	6
**%**	15.4	2.6	7.7	5.1	15.4

**Calcar -** (n = 21)					
**N**	3	1	1	0	4
**%**	14.3	4.8	4.8	0.0	19.0

**Total** (n = 60)					
**N**	9	2	4	2	10
**%**	15.0	3.3	6.7	3.3	16.7

In those patients that were stabilized using a short delta split approach, loss of reduction was significantly higher (2.33 ± 1.99 mm) when compared with those stabilized using a deltopectoral approach (1.08 ± 1.93 mm; p = 0.23). Due to the small number of patients with a minimally-invasive delta split approach (n = 12), however, post-hoc analysis revealed a power of only 0.44 for this statement.

## Discussion

With new minimally-invasive approaches for the angular stable plate osteosynthesis, the need for calcar screws has been discussed increasingly. In order not to harm the axillary nerve some surgeons tend to avoid placement of calcar screws, especially when done percutaneously in minimal-invasive plating. In the present study it was shown that a loss of reduction over time could be prevented by the placement of one or two screws running tangentially to the medial curvature of the humeral surgical neck, commonly referred to calcar screws. It has been suggested that the placement of calcar screws in minimally-invasive approaches increases the risk for lesions to the axillary nerve [35]. In our study, the insertion of calcar screws did not increase the risk of adverse events like damage to the axillary nerve, cut-out, delayed union. Humeral head necrosis occurred similarly in both groups-as far as this conclusion can be drawn with a follow-up of 6 months. Since the rate of humeral head necrosis after locking plates is increasing over time [36], a follow-up of 6 months is too short to draw definitive conclusions about humeral head necrosis. For the evaluation of varus malalignment and consecutive cut-out, however, this time period seems sufficient as the bone-plate-interface plate osteosynthesis of proximal humeral fractures usually fails during the first three, four weeks postoperatively [37].

Loss of fracture reduction was linked to the presence or absence of medial support in locking-plate fixation of proximal humeral fractures by Gardner et al. [29,38]. Yet, this study did not distinguish between anatomic cortical reduction, head-shaft-impaction or an inferomedial screw (analogous to the calcar screw in the present study). In the clinical setup or during surgery, however, it might be difficult to properly evaluate the first two named entities. Moreover, in some cases cortex-to-cortex reduction can result in varus fixation with the clinical problems associated with varus malunions [28].

Even though our findings concerning the measurements of loss of reduction were statistically significant, one has to consider statistical effects associated with the relatively small number of patients. Radiographic loss of reduction indicates humeral varus mal-union, thus resulting in a shorter lever arm of the rotator cuff [39,40] and subacromial impingement due to a reduced acromio-humeral distance [40,41].

The method of measuring the head-plate distance has been described previously [29], but highly depends on a similar humeral rotation on the true AP radiographs. In our institution the latter one is usually defined by rotating the patient 40° towards the affected side, hands lying on the abdomen [42,43]. Due to pain, in some patients it was not possible to rotate the arm accordingly. This implies a considerable variance of humeral rotation and is the main reason urging us to exclude 14 patients from the evaluation of loss of reduction.

We did not take into account bone quality or differences of fracture morphology between the two groups. The complexity of fractures influences the incidence of sustaining nonimplant-related complications [17], and humeral head necrosis is associated with more complex fractures [44] as this is suggested by our data as well (no 2 part fractures were followed by osteonecrosis). In our study, the occurrence of complications (cut-out, axillary nerve lesion, delayed union) and the rate of humeral head necrosis did not differ significantly among the two groups, however.

On the other hand, age and complexity of fractures was higher in group C+, suggesting lower complication rates in the presence of calcar screws.

It is known that the surgical approach to the glenohumeral joint influences the functional but not the radiological outcome [45]. The effect of the surgical approach in the present study is not clear. Seemingly, patients with a short delta-split had higher radiographic loss of reduction. A possible explanation would that the minimally-invasive procedure hardened reduction. However, power of these results is insufficient due to the small number of patients with a delta-split approach. Undoubtedly, no axillary nerve lesions were observed in our study population. Yet, in almost all patients with a delta-split (11/12) the surgeon refrained from placing a calcar screw. Thus, a final statement concerning the influence of the approach on loss of reduction and other complications can not be made.

## Conclusions

The placement of calcar screws in the angular stable plate fixation of proximal humeral fractures is associated with less secondary loss of reduction by providing inferomedial support. An increased risk for cut-out, delayed union or axillary nerve lesion could not be shown. Future studies should consider the importance of medial calcar support.

## Competing interests

The authors declare that they have no competing interests.

## Ethics committee approval

All data was collected according to the terms of reference specified by the local ethics committee http://www.kek.zh.ch/internet/gesundheitsdirektion/kek/de/home.html.

## Authors' contributions

GO participated in designing the study, carried out the radiographical measurements, analysed and drafted the manuscript. CO participated in drafting the manuscript. GW and HPS were involved in the surgical procedures, the classification of the fractures and revised the manuscript. CW participated in designing the study, was involved in the surgical procedures, the classification of the fractures, and the analysis of the data and revised the manuscript. All authors read and approved the final manuscript.
